# Editorial: Cytokines, novel cell death models and pathways in cardiovascular diseases

**DOI:** 10.3389/fcvm.2023.1270320

**Published:** 2023-08-24

**Authors:** Yang-Wei Cai, Mao-Xiong Wu, Qing-Yuan Gao, Jing-Feng Wang, Yu-Li Huang, Yun-Zhao Hu, Ruo-Feng Qiu, Wei-Yi Mai, Hai-Feng Zhang

**Affiliations:** ^1^ Department of Cardiology, Sun Yat-sen Memorial Hospital, Sun Yat-sen University, Guangzhou, China; ^2^ Guangdong Province Key Laboratory of Arrhythmia and Electrophysiology, Sun Yat-sen Memorial Hospital, Sun Yat-sen University, Guangzhou, China; ^3^ Department of Cardiology, Shunde Hospital, Southern Medical University, Foshan, China; ^4^ Capital Health System, Trenton, NJ, United States; ^5^ Department of Cardiology, First Affiliated Hospital of Sun Yat-sen University, Guangzhou, China

**Keywords:** cardiovascular disease, cytokines, cell death, coronary heart disease, ferroptosis

**Editorial on the Research Topic** Cytokines, novel cell death models and pathways in cardiovascular diseases

Cardiovascular disease (CVD) persists as a major global health issue and remains one of the leading causes of mortality worldwide. Cell deaths, especially programmed cell deaths, are critical processes in the development of various CVDs (1). Recently, accumulating studies have shed light on emerging cell death modalities, such as ferroptosis, necroptosis, pyroptosis, PANoptosis, and their relevance to the onset and progression of CVDs (2–4). A comprehensive understanding and targeted exploration of different types of programmed cell death could provide novel insights for the therapeutic targets of CVDs.

Cytokines also play an essential role in CVD development. They are considered to have crucial regulatory roles in CVDs through autocrine, paracrine, and endocrine actions (5, 6). For instance, we have previously reported the important roles of IL-10, sST2, and IL-33 in vascular and myocardial diseases (7–9). Furthermore, many cytokines, such as TNF-α, IL-1β, IL-6, and IL-11 are critically involved in CVD development (6, 10). Importantly, pro-inflammatory cytokines, particularly IL-1β, IFN-γ and TNF-α can directly initiate the cell death program, such as apoptosis and PANoptosis (11, 12). Concurrently, cell death mortalities like pyroptosis and PANoptosis can also promote the release of intracellular components and cytokines, triggering an inflammatory cascade response, thereby contributing to CVDs (13). Research focusing on the crosstalk between cytokines and the cell death pathway may offer novel therapeutic perspectives for heart-related diseases.

Building on this, the research topic “Cytokines, Novel Cell Death Models, and Pathways in Cardiovascular Diseases” published in Frontiers in Cardiovascular Medicine aimed to discuss recent advances and offer insights in this field.

Among the contributions to this special issue, Li et al. presented a comprehensive review on the pivotal role of ferroptosis in CVDs. Ferroptosis, an iron-dependent form of cell death characterized by phospholipid peroxidation, was first identified in 2012 (14). The review by Li et al. delves into the molecular and metabolic mechanisms underlying ferroptosis, including its regulation through lipid oxidation metabolism, glutamate metabolism, and iron metabolism. They summarized the research progress regarding the significance of ferroptosis in various CVD conditions, including arrhythmia, myocardial ischemia-reperfusion injury, atherosclerosis, chemotherapeutic drug-induced cardiotoxicity, heart failure, hypertension, diabetic cardiomyopathy, and septic cardiomyopathy. In addition, the review highlights promising therapeutic strategies targeting ferroptosis in CVDs. Various ferroptosis inhibitors, including ROS inhibitors, iron chelators, and traditional Chinese medicine, have shown potential in mitigating myocardial injury and preserving cardiac function in different CVD scenarios, particularly in myocardial infarction, ischemia-reperfusion injury, and cardiomyopathy. This comprehensive review significantly enhances our understanding of the crucial pathogenic role of ferroptosis in multiple CVD conditions and underscores its promising potential as a therapeutic target for CVDs. Further studies focusing on the regulatory mechanisms and therapeutic applications of ferroptosis in CVDs are urgently warranted.

Diabetic cardiomyopathy is characterized by myocardial dysfunction in diabetic patients, independent of hypertension and structural or coronary heart disease (15). Cardiomyocyte death in metabolic disorders caused by diabetes is a major contributor to the development of diabetic cardiomyopathy. Ke et al. provided a comprehensive review, highlighting the significant roles of ferroptosis, necroptosis, and cuproptosis in the pathogenesis and progression of diabetic cardiomyopathy. They highlighted that targeting these novel regulated cell death pathways could offer potential therapeutic benefits for the treatment of diabetic cardiomyopathy. The review emphasized the need for further researches to explore the similarities and potential overlaps among different regulated cell death pathways to identify optimal drug targets for therapeutic purposes.

Another area of focus in the research topic was coronary heart disease (CHD), a prevalent cardiovascular disorder primarily caused by atherosclerosis and narrowing of the coronary arteries. CHD can lead to severe outcomes such as myocardial infarction, ischemic cardiomyopathy, and heart failure, resulting in significant morbidity and mortality rates. Several studies in this research topic examined different aspects of CHD. Wang et al. evaluated the prognostic value of insulin-like growth factor 1 (IGF-1) and insulin-like growth factor binding protein 2 (IGFBP-2) in patients with acute coronary syndrome (ACS) and found that IGFBP-2 levels were associated with a poor prognosis after ACS. Yu et al. demonstrated that combining Lp(a) levels with carotid intima-media thickness could provide a favorable predictive value for CHD. Wali et al. identified that early atrial remodeling could predict hospitalization for cardiovascular events in patients with new-onset metabolic syndrome. Tong et al. conducted a bioinformatics study and revealed that circRNAs (circRNA0001785, circRNA0000973, circRNA0001741, and circRNA0003922) possess promising predictive capabilities for CHD. Liu et al. conducted a mendelian randomization analysis to investigate the genetic causal relationship between whole-body iron status and CHD development.

In conclusion, this special issue of Frontiers in Cardiovascular Medicine sheds light on the intricate interplay between cell death modalities, cytokines, and their involvement in CVDs. It underscores the importance of further researches on the crosstalk between cell death pathways and cytokine regulation, as it holds significant promise for developing more effective preventive and treatment strategies to address the increasing burden of CVDs worldwide.
